# A rare case of metachronous neuroendocrine tumor after a colorectal adenocarcinoma: qualitative critical review of synchronous and metachronous gastrointestinal NET

**DOI:** 10.1007/s12328-020-01255-9

**Published:** 2020-10-12

**Authors:** Francesco Lancellotti, Luigi Solinas, Davide Telesco, Andrea Sagnotta, Augusto Belardi, Giuseppina Balsamo, Stefano Mancini

**Affiliations:** 1 Department of Surgical Sciences, Sapienza University of Rome, Rome, Italy; 2 Department of General Surgery and Surgical Oncology, San Filippo Neri Hospital, Rome, Italy; 3 Department of General Surgery, Surgical Specialities “Paride Stefanini”, Sapienza University of Rome, Rome, Italy; 4 Department of Clinical Pathology, San Filippo Neri Hospital, Rome, Italy

**Keywords:** Metachronous neuroendocrine, Synchronous NET, Colorectal adenocarcinoma, Coexisting tumors, Gastrointestinal carcinoids

## Abstract

Gastrointestinal neuroendocrine tumor (NET) associated with a metachronous intestinal adenocarcinoma is rare. We report the case of a 71-year-old man with an ileal NET. Patient has previously undergone a left colectomy for sigmoid cancer. We report a complete review both of the metachronous and synchronous NET. A comprehensive systematic literature search in PubMed, EMBASE, and MEDLINE identified a total of 35 relevant studies. This study includes an analysis of review articles, case reports, case series, retrospective studies and population-based studies. In the English literature to date, there are 21 case reports (19 synchronous cases and 2 metachronous cases), 3 case series and 3 review articles, and less than 10 retrospective studies or population-based studies. A total of 31 patients in 24 articles were included in the study: 28 patients with a synchronous gastrointestinal NET and colorectal adenocarcinoma and 3 patients with metachronous gastrointestinal NET and colorectal adenocarcinoma. The incidence of synchronous cancer (particularly for colorectal and gastric cancer) with a gastrointestinal NET ranges from 10 to 50%, while for the metachronous ones it is still unclear. This is the third metachronous case report and the first descriptive case of gastrointestinal NET diagnosed 2 years after a colorectal adenocarcinoma. An endoscopic follow-up program for gastrointestinal NET patients and/or for first-degree relatives of NET patients appears recommendable.

## Background

Neuroendocrine tumors of the small intestine (ileal NETs) represent the most common small-bowel neoplasms (31–41% of all small-bowel malignancies), 13% of all NETs and 40% of the gastrointestinal NETs [1, 2]. The incidence is estimated at about 1–2 cases/100,000 inhabitants/year [3].

The most frequent NET location is the terminal part of the small intestine [4], in particular the last 60 cm, and in 33% of the cases they are multiple along with the tenuous [5]. They can remain asymptomatic until the appearance of metastases, or they can cause anemia and/or obstructive symptoms, from transient abdominal pain to occlusion due to stenosis or intestinal intussusception. The survival at 5 years after diagnosis of NET of the non-metastatic small intestine is 65% [4].

Several population-based studies show that patients with gastrointestinal NETs may have an associated metachronous primary tumor and vice versa [6]. The literature reports only two cases of metachronous NET.

We describe herein the third case of metachronous ileal NET and report a complete review both of the metachronous and synchronous NET.

### Case presentation

A 71-year-old Caucasian man with a significant past medical history for cholecystectomy, ex-smoker, obese (BMI 32.4) came to the emergency room with a 2-week history of intermittent pain in the left quadrant of the abdomen. Flexible colonoscopy was performed for further evaluation that showed large stenosing polyps in the sigmoid. Pathological finding revealed dysplasia associated with differentiated invasive adenocarcinoma. CT abdomen showed sub-stenosing mass in the left colon; no lesions in the small bowel and/or hepatic nodules were revealed.

**Fig. 1:**
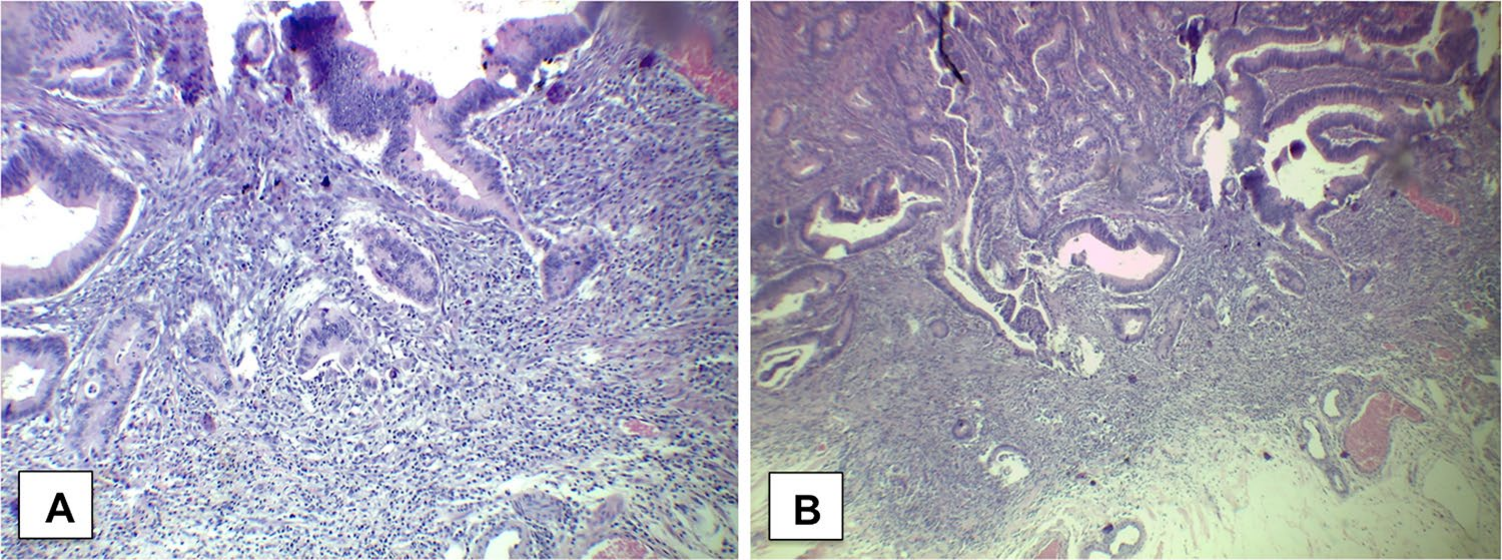
Photomicrographs of resected specimen (adenocarcinoma). Note the foci of adenocarcinoma associated with high-grade tumour budding in **a** (haematoxylin and eosin × 40). In **b**, magnification × 100 showing a component of villous tubular adenoma with low-grade dysplasia, focally high-grade (haematoxylin and eosin).

Thus, he underwent left colectomy for sigmoid neoplasm in June 2018. The histologic examination revealed a villous tubular adenoma with low-grade dysplasia, focally high-grade with foci of adenocarcinoma associated with high-grade tumour budding (UICC 2012: pT1 pN0 M0) (Fig. 1).

In January 2020, the patient was admitted to our Department of General Surgery with a 3-week history of sub-occlusive episodes with nausea, recurrent episodes of constipation and epigastric abdominal pain.

CT scan showed hydro-aero levels in the upper abdominal and mesogastric quadrants, and last loops grouped by thickened walls with a 3 cm solid nodule with multiple small surrounding reactive lymph nodes.

**Fig. 2:**
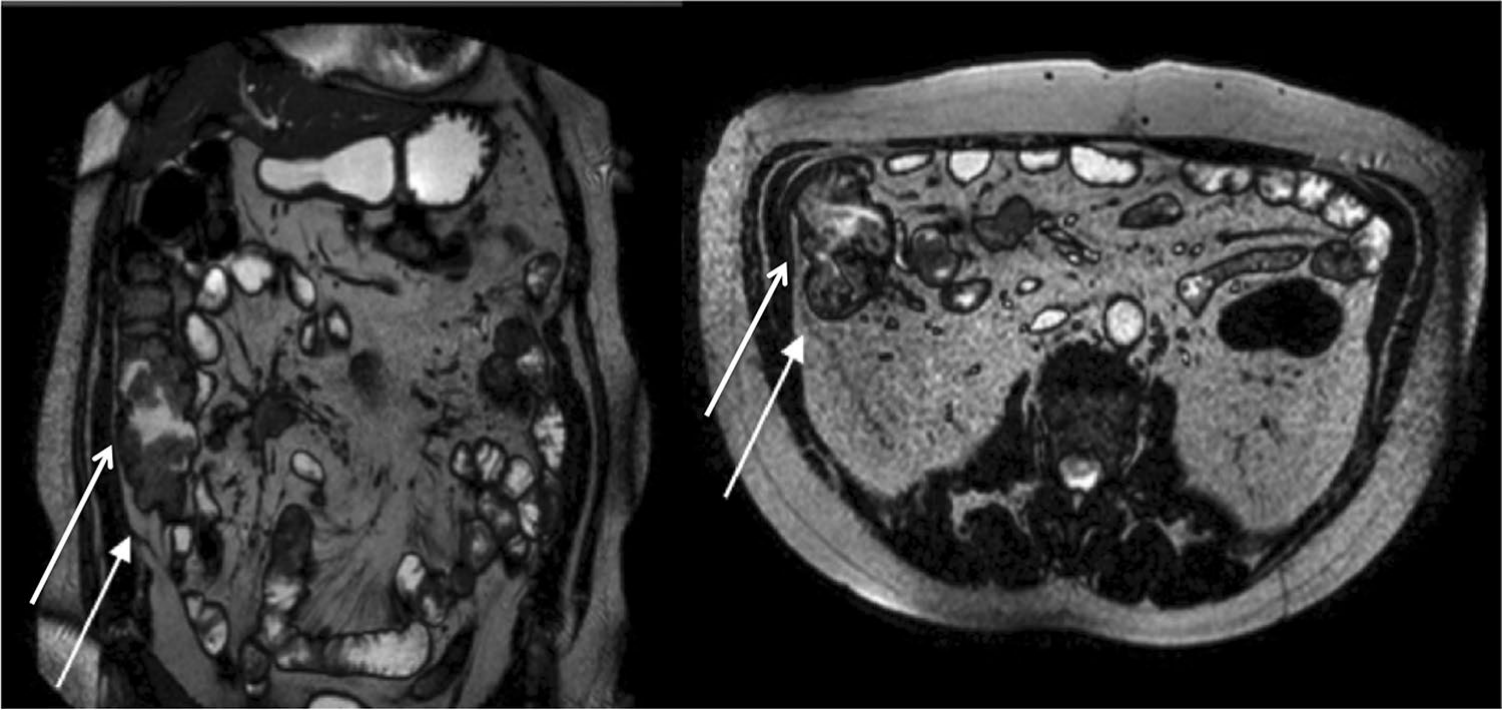
Pre-operative MRI shows a nodule within the wall of the distal ileal (arrow) and within the mesentery. Coronal (left) and axial plane (right)

The entero-MRI showed a 3 cm-vascularized formation within the mesentery not in contact with tenuous loops, and two vascularized locations of 2.6 cm and 1.6 cm within the wall of the distal ileal tract (Fig. 2).

**Fig. 3:**
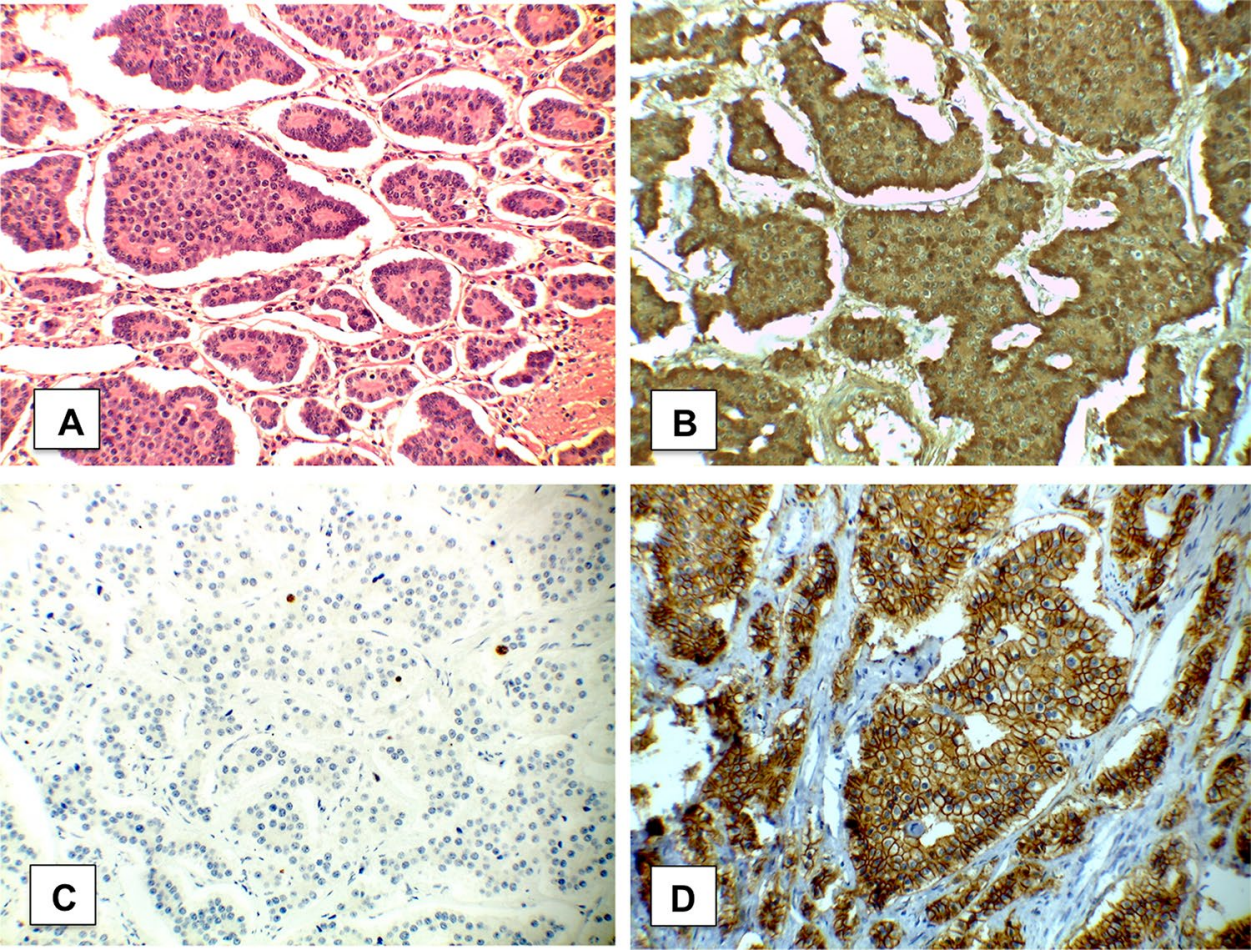
Photomicrographs of the resected specimen. Note the organoid and trabecular patterns of the NET cells (**a**), (haematoxylin and eosin staining, original magnification × 400). Chomogranin stains of the resected NET (**b**). Antigen ki-67 immunostaging was positive in 3% of tumor cells (**c**). Positivity for CD56 in photo **d**

At the operation, the wall of the ileal loop was thick and fibrous with numerous tenacious viscera-wall and viscera-viscera adhesions. A viscerolysis and an ileal resection were performed. Patient was discharged in 7 POD with an unremarkable post-operative course. The histologic examination (Fig. 3) revealed a well-differentiated neuroendocrine tumour of the distal ileum (carcinoid, NET G1 sec. WHO 2010) pT3-stage I sec. UICC 2009) G1 pT3 pN2 pMx sec AJCC 8TH edition 2017. Ki67/MIB-1 < 3%, CK7−; CK20−; CD56+; NSE+; Synaptophysin+; Chromogranin A+.

## Methods

A comprehensive systematic literature search was carried out in PubMed, EMBASE, and MEDLINE to identify relevant articles. The MeSH terms were “NET”, “neuroendocrine tumor”, “carcinoid”, and “small intestinal carcinoids”, combined with MeSH terms “colorectal adenocarcinoma”, or “colorectal tumor” as well as “colonic tumor”, “synchronous” and “metachronous”. The relevant reference lists of articles were also searched manually for additional works. Two different researchers carried out the search independently. The last search was performed in the first half of April 2020. Articles were limited to manuscript publications in the English language and/or all abstract publications. A total of 35 relevant studies were found and examined. This study includes an analysis of review articles, case reports, case series, retrospective studies and population-based studies.

### Results

To date, the literature in English reveals, 20 case reports (18 synchronous cases [7–24], 2 metachronous cases [25, 26]), 2 case series [25, 27], 3 review articles [28–30], and about 10 retrospective studies [31–34] or population-based studies from a National Registry [35–41] of synchronous or metachronous NET with a second primary malignancy (SPM). A total of 31 patients in 24 articles were included in the study: 28 patients with a synchronous gastrointestinal NET and colorectal adenocarcinoma (Table 1) and 3 patients (including the present study) of 3 articles with metachronous gastrointestinal NET and colorectal adenocarcinoma (Table 2).

**Table 1:** Literature review of synchronous cases with both gastrointestinal adenocarcinoma and gastrointestinal NET

Study	Year	Age	Sex	Carcinoma location	Net location	Grading
Pearson and Fitzgerald [27]	1949	88	M	Left colon	Small bowel	–
Pearson and Fitzgerald [27]	1949	73	M	Sigmoid colon	Stomach	–
Pearson and Fitzgerald [27]	1949	61	F	Left colon	Small bowel	–
Khubchandani et al. [7]	1972	53	M	Rectum	Rectum	–
Lotlikar et al. [25]	1981	53	M	Rectum	Small bowel	–
Lotlikar et al. [25]	1981	67	F	Right colon	Small bowel	–
Lotlikar et al. [25]	1981	60	F	Sigmoid colon	Small bowel	–
Sacchi et al. [8]	1988	57	M	Right colon	Small bowel	–
Tse et al. [9]	1997	72	M	Hepatic flexure	Small bowel	–
Habal et al. [28]	2000	52	M	Sigmoid colon	Rectum	
Cioffi et al. [10]	2005	64	F	Ileum	Small bowel	–
Klucinski et al. [11]	2006	72	F	Transverse colon	Small bowel	–
Chemli et al. [12]	2007	63	F	Right colon	Small bowel	–
Aslam et al. [13]	2009	67	F	Sigmoid colon	Small bowel	G1
Boland et al. [14]	2009	77	F	Left colon	Meckel's diverticulum	–
McHugh et al. [15]	2009	74	F	Rectum	Small bowel	–
Wohadlo et al. [29]	2011	53	M	Splenic-hepatic flexure	Small bowel	–
Gurzu et al. [16]	2012	78	F	Sigmoid colon	Small bowel	G3
Pozzato et al. [17]	2012	61	M	Right colon	Duodenum	G1
Katalinic 2014 et al. [18]	2014	63	M	Right colon	Meckel's diverticulum	G1
Zhu et al. [19]	2015	64	F	Rectum	Rectum	G1
Almajano et al. [20]	2016	66	M	Right colon	Small bowel	G1
Mohapatra et al. [21]	2016	83	M	Sigmoid colon	Left colon	G3
Nejatollahi et al. [22]	2016	83	M	Sigmoid colon	Small bowel	G1
Vootla et al. [23]	2016	46	F	Hepatic flexure	Rectum	G1
Winn et al. [30]	2017	40	M	Sigmoid colon	Rectum	G1
Winn et al. [30]	2017	70	M	Sigmoid colon	Rectum	G1
Napolitano et al. [24]	2017	72	M	Right colon	Small bowel	G1–G2

*NET* neuroendocrine tumour

**Table 2:** Literature review of metachronous cases with both gastrointestinal adenocarcinoma and gastrointestinal NET

Study	Age	Sex	Carcinoma location	Net location	Presentation	Time interval (years)
Lotlikar et al. [25]	38	M	Right colon	Small bowel	NET before	4
Fujimoto et al. [26]	90	M	Sigmoid colon	Rectum	Carcinoma before	2
Present study	71	M	Sigmoid colon	Small bowel	Carcinoma before	2

*NET* neuroendocrine tumour

Mean patient age was 65.5 years (range 38–90 years), by gender there were 12 females (39%) and 19 males (61%), undergoing gastrointestinal surgery.

The adenocarcinoma location was: sigmoid colon (35.5%, 11 patients), right colon (25.8%, 8 patients), rectum (12.9%, 4 patients), left colon (9.7%, 3 patients), hepatic flexure (6.5%, 2 patients), splenic flexure (3.2%, 1 patient), transverse colon (3.2%, 1 patient), and small bowel (3.2%, 1 patient). In all the cases the tumor was resectable. NET location was: small bowel (67.8%, 21 patients), rectum (22.6%, 7 patients), left colon (3.2%, 1 patient), stomach (3.2%, 1 patient) and duodenum (3.2%, 1 patient).

In all synchronous cases, symptoms and signs were related to the adenocarcinoma, while NET was often asymptomatic.

### Discussion

We performed a comprehensive systematic literature search was carried out and summarized the reported cases with both synchronous gastrointestinal NET and colorectal adenocarcinoma in Table 1, and the metachronous ones in Table 2. The case reports of synchronous tumors are more common than metachronous ones. In all cases, symptoms and signs were probably related to the adenocarcinoma instead of NETs. The tumors were in all cases resectable. The most frequent carcinoma location was the sigmoid colon, while the most frequent small intestinal NET location was the small bowel. In three cases, the tumor was multifocal: in two cases [9, 15] there were two foci of small intestinal NETs in the small bowel, in one case [29] there were two small intestinal NETs in the small bowel and two carcinomas in the colon. Pearson and Fitzgerald were the first to report the high incidence (23%) of second primary malignancies (SPM) in patients with small intestinal NETs at autopsy [27]. A certain number of NETs were incidentally found during surgery for other cancers. The estimated rate of SPM associated with other malignancies was 2.3% in surgical cases and 8.1% in autopsy cases [42].

The literature in English contains several reviews. Habal et al. collected a large series of cases: over 5000 cases in about 14 articles, including autopsy studies and cases collected by files at Cancer registry resources [28]. They found that small-bowel small intestinal NETSs had the highest rate of SPM (29–52%), followed by appendiceal (13–32%) and colorectal small intestinal NETs (5–32%), and that most of SPM and small intestinal NETs were synchronous (59–87%). In a report from the SEER database (Surveillance, Epidemiology and End Results Program, National Cancer Institute, USA), 29% of patients with gastrointestinal small intestinal NET had an additional malignancy [41].

The first studies investigating the incidence of additional primary cancers among patients with NETs were autopsy studies (31, 36), while in recent years population-based studies in the National Registry have increased [35–41]. Zar et al. used the Swedish Cancer Registry to estimate excess risk of second primary malignancies among 3741 small intestinal NET (SINET) patients [37].

A very interesting article was published by Amin et al.: it is the first study that used the US-based SEER database in the USA, and the first to quantify prognoses and predictors of additional malignancies in SINET patients [38]. They also quantified the risk of developing SINET, both future primaries after SINET (post-SINET) as well as the risk of future SINET among patients with non-SINET primaries (pre-SINET).

Kamp et al. and Clift et al. found that the only significantly elevated risk of SPM was for synchronously diagnosed neoplasms [32, 33]. There were no statistical differences between observed and expected occurrences of SPM in their previous and metachronous subsets.

In 2014, Kauffmann et al. conducted a population-study using the Surveillance and Epidemiology, and End Results (SEER) database, an even bigger one [40]. They identified a cohort of 9727 patients with pancreatic NET (PNET) or gastrointestinal NET (GINET) and found an incidence of additional malignancies of 25.8%. Patients with GINET had an increased risk of additional malignancies, particularly for colorectal and gastric cancer, whereas patients with PNET had a decreased risk of a second malignancy compared with the general population.

Thus, synchronous malignancies with NETs are more common than metachronous cases [28, 38, 43], but metachronous malignancies can occur anywhere [28, 39, 44]. Metachronous tumors can occur 1–7 years after the NET is diagnosed [45]. Except for the autopsy studies and population-based study, in the literature in English there are only two case reports about metachronous NET with a SPM [25, 26]. In our study, we present the third metachronous case report and the first descriptive case of gastrointestinal NET diagnosed 2 years after a colorectal adenocarcinoma.

Concerning the prognosis, Amin et al. demonstrated that patients diagnosed with a carcinoma before their SINET (subgroup pre-SINET) have a worse prognosis than patients diagnosed with SINET as a cancer of first diagnosis (subgroup post-SINET), mean survival 57.9 vs 40.9 months [38]. Several studies agree that the overall prognosis depends primarily on the more aggressive SPM [16, 28]. In fact, in only one of the 270 cases analyzed by Berner, the NET itself changed the prognosis [46].

Many hypotheses have been advanced to explain the pathogenesis of association between NETs and second primary malignancy: a genetic predisposition, common environmental exposures or behavioral risk factors, exogenous mitogenic effects of secretory products from a primary tumor causing neoplastic transformation, or a combination of all these factors [41, 47]. The population-based study by Kharazmi et al., using the nationwide family cancer data sets of Sweden and Finland, found that first-degree relatives of patients with gastrointestinal NET had an increased risk of developing these tumors, thus suggesting a potential counseling for this population [48]. Ito et al. [49] conducted a nationwide survey to examine the epidemiology of GINETs in Japan, showing that incidence of new-onset PNET in 2005 was approximately 1.01 per 100,000 population in Japan, which was approximately three times the annual incidence of new-onset PNET in the United States. Five years later, the same group clarified some epidemiological changes [50]: they found that incidences of GINET in Japan were lower than those reported in Western nations, but similar than those reported in China, Taiwan and Korea, suggesting ethnic differences.

In 2013, Shenoy described a case series of 11 patients with gastrointestinal cancers and synchronous NET, but no metachronous tumors. All patients presented a pattern of similar embryonic visceral origin, thus suggesting that SINET may produce growth factors and exert a paracrine effect that may increase a person’s predisposition to local colorectal adenocarcinoma [51]. SINETs also produce non-neuroendocrine peptides that may play a role in carcinogenesis as growth factors. Their principal role is to regulate cell growth and differentiation [52, 53]. Recently, PDGF, EGF, TGF, insulin-like growth factors, and FGF have been demonstrated in gastrointestinal NETs [54], and these growth factors may play a central role in the genesis of SPMs in patients with NETs. In fact, over 30 other peptides have been isolated from neuroendocrine cells. It is plausible that many of these peptides may play similar roles in tumorigenesis. It is also possible that the increased risk of a second cancer in patients with gastrointestinal NET is related to common genetic or tumorigenic pathways. There is a growing body of literature that reports an increased incidence of other malignancies in patients with gastrointestinal NETs in absence of a known genetic syndrome. This may indicate still-unknown genetic mutations predisposing to multiple malignancies [28, 39]. Finally, the interaction between environmental and genetic factors and/or treatment-related or lifestyle factors such as smoking and alcohol may promote the carcinogenesis of second cancers after NETs [35].

This qualitative and critical review underscores the importance of increased surveillance for other malignancies in patients with gastrointestinal NET. Although enrolment in follow-up programs (such as colonoscopy or gastroscopy) cannot be ascertained from the SEER database, case reports or article reviews, a closer surveillance than that of the general population can be recommended for NET patients and/or first-degree relatives of NET patients.

### Conclusion

In our study, we present the third metachronous case report and the first descriptive case of gastrointestinal NET diagnosed 2 years after a colorectal adenocarcinoma. In retrospective and population-based studies, the incidence of synchronous cancer (particularly for colorectal and gastric cancer) with a gastrointestinal NET range from 10 to 50% is found, for the metachronous’ cancer it is still unclear if there is a direct connection or a genetic predisposition. The underlying pathogenesis may be a combination of factors, such as genetic predisposition, common environmental exposure or behavioral risk factors and/or exogenous mitogenic effects of secretory products from a primary tumor. Therefore, at the present state of the art, it is impossible to draw up guidelines on this issue, but an endoscopic follow-up program for gastrointestinal NET patients and/or for first-degree relatives of NET patients appears recommendable for early detection of a synchronous second gastrointestinal cancer. However, this study does not support extensive screening programs for metachronous second primary malignancies in NET patients.
